# Diagnostic pitfalls of varicella virus infection in adults

**DOI:** 10.1186/1471-2334-13-S1-P70

**Published:** 2013-12-16

**Authors:** Alina Demetrian, Gabriela Iliescu

**Affiliations:** 1 Emergency County Hospital Târgu Jiu, Romania; 2 Clinical Emergency County Hospital Craiova, Romania

## Background

Usually, varicella in adults has a more severe evolution than in children, with a higher incidence of complications.

## Case report

We present three cases of previously healthy adult patients diagnosed with varicella in December 2012 with uncommon onsets, but with mild evolution of the disease.

Patient 1: female, age 45, had an atypical onset with very few vesicles - only one on the trunk, and then on the head after five days, but with inflammatory occipital lymph nodes. From day 6, our patient had typical dermatological lesions, mild pruritus and only two days of mild fever.

Patient 2: male, age 38, had a typical onset, with high fever in the first week. With another episode of varicella in his childhood, this was considered as a varicella virus recurrent infection.

Patient 3: male, age 80, was admitted to the hospital for extremely painful cutaneous eruptions on the thorax, initially diagnosed as zona zoster. In the next days, the lesions, in different stages of development, extended all over the body, with typical evolution of chickenpox.

All patients were treated with supportive therapy: anti-pruritic and anti-inflammatory drug mouthwashes, soothing topical lotions and anesthetic gels. They had self-limited evolution, with no complications and with complete remission of lesions.

## Conclusion

The incidence of chickenpox in adults seems to be higher in last years. As varicella-zoster virus infection can present unusual manifestations, clinicians must be aware of these presentations and be prepared to perform appropriate diagnostic and treatment.

